# Antibacterial Activity of Terpenes and Terpenoids Present in Essential Oils

**DOI:** 10.3390/molecules24132471

**Published:** 2019-07-05

**Authors:** Aline Cristina Guimarães, Leandra Martins Meireles, Mayara Fumiere Lemos, Marco Cesar Cunegundes Guimarães, Denise Coutinho Endringer, Marcio Fronza, Rodrigo Scherer

**Affiliations:** 1 Pharmaceutical Sciences Graduate Program, Universidade Vila Velha, Espírito Santo 29102-770, Brazil; 2 Department of Morphology, Federal University of Espírito Santo, Espírito Santo 29043-090, Brazil

**Keywords:** essential oil, terpenes, bacteria, time kill kinetics, antimicrobial activity, bactericidal

## Abstract

**Background:** The antimicrobial activity of essential oils has been reported in hundreds of studies, however, the great majority of these studies attribute the activity to the most prevalent compounds without analyzing them independently. Therefore, the aim was to investigate the antibacterial activity of 33 free terpenes commonly found in essential oils and evaluate the cellular ultrastructure to verify possible damage to the cellular membrane. **Methods:** Screening was performed to select substances with possible antimicrobial activity, then the minimal inhibitory concentrations, bactericidal activity and 24-h time-kill curve studies were evaluated by standard protocols. In addition, the ultrastructure of control and death bacteria were evaluated by scanning electron microscopy. **Results:** Only 16 of the 33 compounds had antimicrobial activity at the initial screening. Eugenol exhibited rapid bactericidal action against *Salmonella enterica* serovar Typhimurium (2 h). Terpineol showed excellent bactericidal activity against *S. aureus* strains. Carveol, citronellol and geraniol presented a rapid bactericidal effect against *E. coli*. **Conclusions:** The higher antimicrobial activity was related to the presence of hydroxyl groups (phenolic and alcohol compounds), whereas hydrocarbons resulted in less activity. The first group, such as carvacrol, l-carveol, eugenol, *trans*-geraniol, and thymol, showed higher activity when compared to sulfanilamide. Images obtained by scanning electron microscopy indicate that the mechanism causing the cell death of the evaluated bacteria is based on the loss of cellular membrane integrity of function. The present study brings detailed knowledge about the antimicrobial activity of the individual compounds present in essential oils, that can provide a greater understanding for the future researches.

## 1. Introduction

Essential oils consist of a complex mixture of compounds, usually from 20 to 60, at different concentrations [1]. Terpenes, the main constituents of essential oils, are derived from the isoprenoid pathway, and are produced and secreted from specialized plant tissues [2]. They are composed of isoprene units (C_5_), which is the basis for their classification, i.e., two isoprene units form monoterpenes (C_10_), three units form sesquiterpenes (C_15_), four units form diterpenes (C_20_), six units form triterpenes (C_30_) and eight units form carotenoids (C_40_) [3]. Terpenes can have several different chemical functionalities, including alcohol (linalool, geraniol, carveol, citronellol, terpineol, menthol, borneol, and bisabolol), aldehyde (citral and citronellal), phenol (thymol and carvacrol), ketone (carvone and camphor), ether (eucalyptol) and hydrocarbon (cymene, pinene, limonene, and phellandrene) groups. 

The evaluation of the biological activity of essential oils has been carried out over the years in order to identify new compounds with antibacterial activity for industrial applications [4,5,6]. The food industry has been looking for natural compounds, since the use of synthetic antimicrobial additives cause cancer in several tissue types. For example, parabens cause breast cancer [7,8] and nitrites cause cancer in lung, intestine, liver and stomach [9]. In the medical area, the search for new compounds becomes more and more urgent, as the emergence of resistant pathogens compromises therapy and raises the mortality rate. *Staphylococcus aureus, Salmonella* spp., *Escherichia coli* and *Bacillus cereus* are important example of bacteria that affect the quality and safety of food [10,11]. 

*S. aureus* and *B. cereus* (Gram-positive) are related to food poisoning due to ingestion of food containing enterotoxins, for example staphylococcal enterotoxin and non-hemolytic enterotoxin, respectively. Several countries report cases of food poisoning caused by *S. aureus* [12,13,14,15]. In addition, *S. aureus* is responsible for a broad spectrum of diseases ranging from superficial skin infections to systemic infections. *B. cereus* is recognized for serious cases of food poisoning, it is also involved in cases of pneumonia, bacteremia and meningitis in immunocompromised patients [16,17,18]. In addition, due to its ability to form spores and structure resistant to high temperatures, it presents a significant risk to food safety, as it survives food processing techniques, such as pasteurization [11].

*E. coli* (Gram-negative) presents pathotypes responsible for extraintestinal infections such as urinary tract infections and diseases associated with food poisoning. There are six *E. coli* pathotypes including: enterotoxigenic *E. coli* (ETEC), enteropathogenic *E. coli* (EPEC), enteroaggregative *E. coli* (EAEC), enterohemoragic *E. coli* (EHEC), enteroinvasive *E. coli* (EIEC), diffusely adherence *E. coli* (DAEC) [19]. *Salmonella enterica* serovar Typhimurium (Gram-negative) is another agent responsible for intestinal infection associated with ingestion of contaminated food, mainly through the consumption of poultry and eggs. Inadequate hygiene conditions is the main cause of *S.* Typhimurium infection, typhoid fever is the most aggressive form of the disease. It is believed that the incidence of infections is underestimated, since several cases are misdiagnosed or are not reported [20].

The risk to human health generated by food contamination, as well as the need for compounds with antimicrobial action, low toxicity, and low cost, encourages the search for new compounds. The differential of the present study is the evaluation the activity of 33 terpenes, including minority compounds, helping to understand the antimicrobial activity of essential oils. In this context, the objective of the present study was to investigate antibacterial and bactericidal activities of terpenes frequently reported in the secondary metabolism of plants, as well as the time of death of bacteria caused by these terpenes. In addition, the ultrastructure was evaluated to confirm cellular changes that may have occurred.

## 2. Results

### 2.1. Screening

The antimicrobial activity was first evaluated by initially screening the compounds at a concentration of 0.25 mg/mL to exclude compounds without activity from the study. Of the thirty-three compounds evaluated, seventeen did not exhibit positive effects against any of the bacterial strains tested, and sixteen compounds showed activity (Table 1). 

### 2.2. Minimum Inhibitory Concentrations

The results of the MIC determination are presented in Table 2. The negative control was considering drug free, which showed the strains viability and to the positive control was used sulfanilamide. The classification of the antimicrobial actions of pure compounds is not well consolidated in the literature. Thus, the comparison with previous results is difficult, since many variables can affect the final result.

The results of the present study show that of the sixteen compounds with antibacterial activity, thymol, carvacrol and eugenol presented strong antimicrobial action against the four strains evaluated even higher than sulfanilamide. On the other hand, the compounds *m*-cymene, (±)-linalool, camphor, (+)-borneol and *R*-(+)-limonene demonstrated the least action against these evaluated strains. Thymol and eugenol inhibited (IC_100_) *S.* Typhimurium and *S. aureus* growth, and were considered potent antimicrobials according to the literature [21].

### 2.3. Minimum Bactericidal Concentration

Only six of the sixteen compounds that showed antimicrobial activity showed bactericidal activity (Table 3). None of the evaluated compounds had bactericidal activity (absence of growth) against *B. cereus*. For *S. aureus*, only thymol and terpineol showed bactericidal activity at the concentration of 0.12 mg/mL. Eugenol presented the lowest MBC value against *S.* Typhimurium (0.06 mg/mL). Thymol was the compound that exhibited the lowest MIC values against the strains evaluated; however, thymol did not have bactericidal activity at concentrations of MIC, 2× MIC and 4× MIC, but the bactericidal activity of thymol was verified at 0.12 mg/mL, as shown in Table 3. 

### 2.4. Time-kill Curve Studies

**Figure 1:**
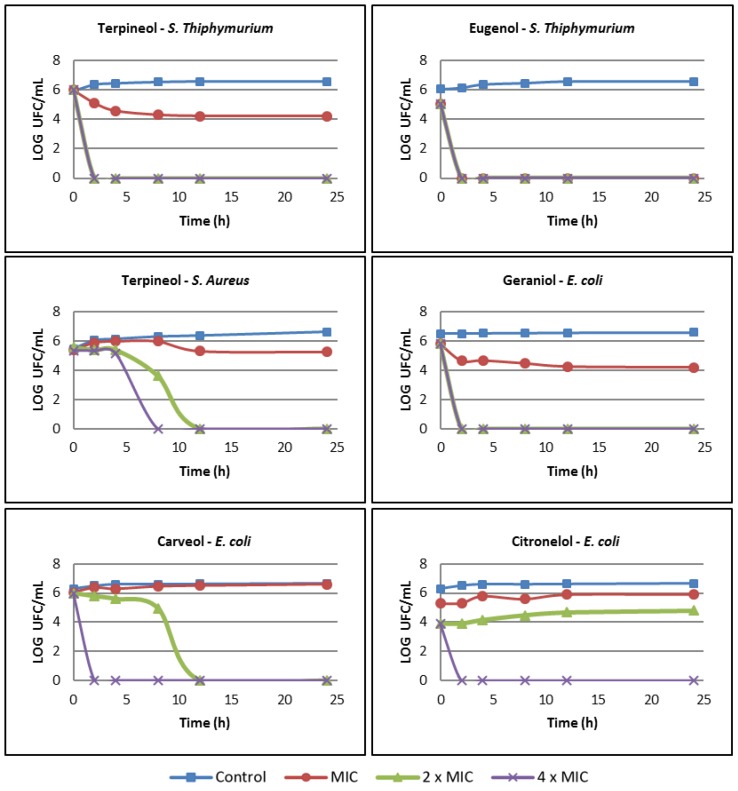
Time-kill curves for the bacteria *S.* Typhimurium, *S. aureus* and *E. coli*. Control: bacteria untreated; MIC: minimum inhibitory concentration obtained by the MIC assay to each terpene.

The time-Kill curve analysis was used to characterize the bactericidal action, in this way only compounds that showed minimal bactericidal activity were evaluated. Figure 1 shows the results of five compounds killing bacterial strains *S.* Typhimurium, *S. aureus* and *E. coli* as a function of time. The results showed that the kinetics of the compounds killing the three bacterial strains were concentration dependent, with higher concentrations leading to faster bacterial death, revealing a dose-dependent effect.

Terpineol and eugenol presented bactericidal action against *S.* Typhimurium, where eugenol caused the death of the bacteria at all the evaluated concentrations in only 2 h. On the other hand, the MIC of terpineol reduced the number of CFUs by only 2 log_10_ in 24 h, and terpineol was not considered bactericidal at this concentration; however, at 2× MIC and 4× MIC, the number of CFUs was reduced by 6 log_10_ in only 2 h. *S. aureus* was only weakly influenced by terpineol at its MIC since the number of cells was close to the control count. On the other hand, at 2× MIC and 4× MIC, the killing time was significantly reduced (12 h and 8 h, respectively). l-Carveol, β-citronellol and *trans*-geraniol were not effective at killing *E. coli* at the MIC. On the other hand, these three compounds rapidly killed bacteria at 4× MIC; carveol and geraniol decreased the number of CFUs by 6 log_10_ in 2 h, and citronellol decreased them by 4 log_10_.

### 2.5. Scanning Electron Microscopy

**Figure 2:**
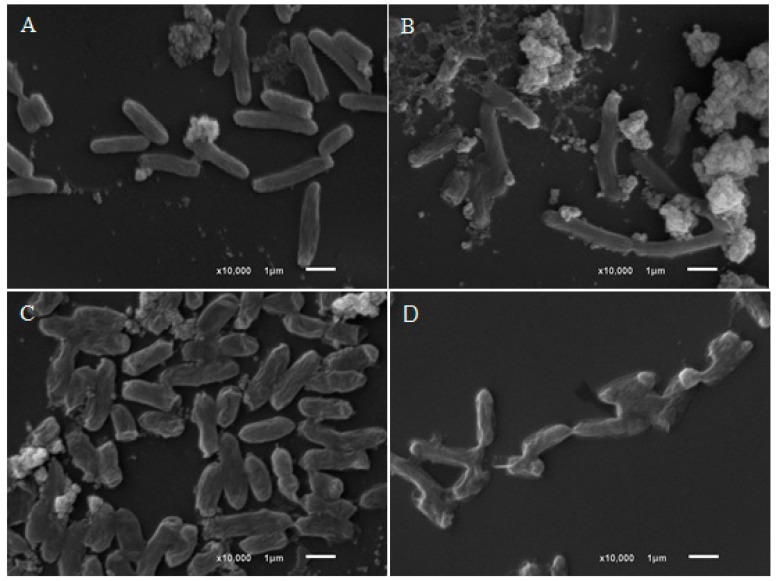
Scanning Electron Microscopy of untreated *E. coli* (**A**) and *E. coli* treated with carveol (**B**), citronellol (**C**) and geraniol (**D**). **A**: SEM of untreated *E. coli* strains; **B**: *E. coli* strains treated with 4× MIC of carveol; **C**: *E. coli* strains treated with 4× MIC of citronellol; **D**: *E. coli* strains treated with 4× MIC of geraniol.

The morphological changes resulting from treatment with l-carveol, β-citronellol and *trans*-geraniol were observed in *E. coli, S. aureus* and *S.* Typhimurium by SEM. The *E. coli* control cells (Figure 2) had bacillary forms and smooth surfaces, whereas the treated cells were irregularly sized with the presence of debris, possibly by disrupting cell division or dysfunction of cellular membrane. 

**Figure 3:**
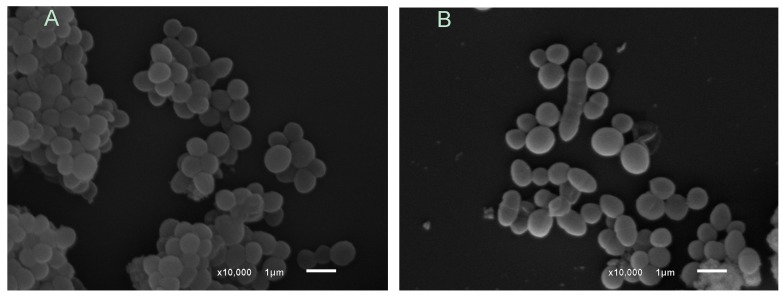
Scanning Electron Microscopy of untreated *S. aureus* (**A**) and *S. aureus* treated with terpineol (**B**). **A**: SEM of untreated *S. aureus* strains; **B**: *S. aureus* strains treated with 4× MIC of terpineol.

Cells treated with citronellol and geraniol were smaller, had significantly rough surfaces and adhered to each other. The treatment of *S. aureus* (Figure 3) with terpineol interrupted cell division and altered the "grape bunch" morphology, a typical form of the colonies. 

**Figure 4:**
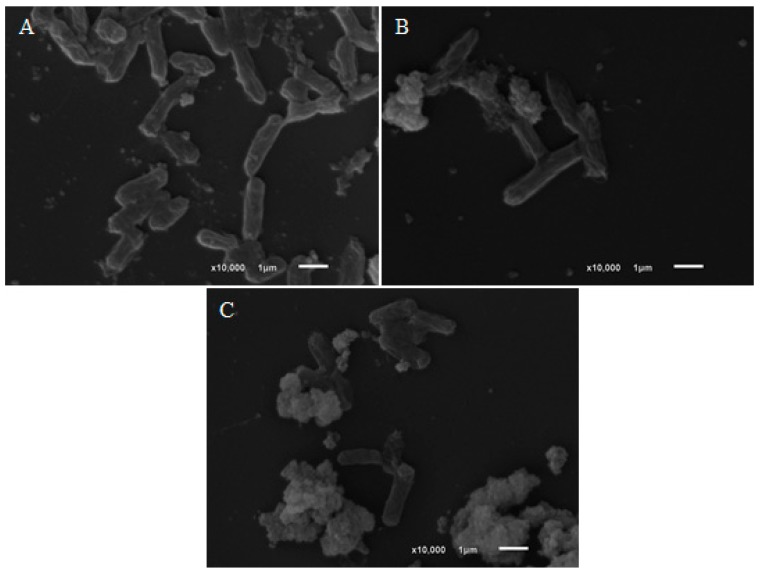
Scanning Electron Microscopy of untreated *S.* Typhimurium (**A**) and *S.* Typhimurium treated with eugenol (**B**) and terpineol (**C**). **A**: SEM of untreated *S.* Typhimurium strains; **B**: *S.* Typhimurium strains treated with 4× MIC of eugenol; **C**: *S.* Typhimurium strains treated with 4× MIC of terpineol.

Treatment of *S.* Typhimurium (Figure 4) with eugenol and terpineol indicate that the mechanism of the death is due to loss of cellular membrane integrity or function, where the cell membrane were completely destroyed, with presence of cell debris.

## 3. Discussion

The major constituents of the essential oils may constitute up to 85%, while other constituents are present in trace amounts [1]. The great majority of studies that evaluate the antimicrobial activity of essential oils leave a gap in the literature since they report difficulty in assigning activity to major compounds or synergism between compounds, in this way it is possible that compounds in smaller amounts also contribute to the activity. The present work thus brings relevant data that will contribute to further studies of the antimicrobial activity of essential oils and to applications in industrial fields, such using these compounds as alternatives to additives in the food industries. 

Factors determining the activity of essential oils are composition, functional groups present in active components, and their synergistic interactions [22]. Previous studies state that oxygenated terpenes (terpenoids) such as phenolics exhibit better antimicrobial activity than hydrocarbons such as *R*-(–)-limonene, terpinene, camphene, and (+)-α-pinene, which agree with the present work, since these compounds presented weak antimicrobial action [22,23,24,25,26]. The results of the present study agree that oxygenated functional groups in terpenes compounds exhibited better antimicrobial activity than hydrocarbons. Previous study reported MIC of 0.031 mg/mL for thymol and 0.015 mg/mL carvacrol against *S. aureus* [27]. In the present study similar values were found, the MIC was 0.007 mg/mL for thymol, and 0.015 mg/mL for carvacrol against *S. aureus*. According to Mazarei et al. [28], the MICs for *E. coli* strain and *S. aureus* were 0.25 and 0.125 mg/mL, respectively, for carvacrol. As mentioned before, the classification of the antimicrobial actions of pure compounds is not well consolidated in the literature making difficult the comparison with previous results. In addition, this difficulty has been reported in previous studies [29,30].

Another relevant point of the antimicrobial activity is the mechanism of action. It can vary with the type of the essential oil or the strain of the microorganism used [22]. Previous studies have proposed that aromatic nuclei with a polar functional group are responsible for antimicrobial activity, but the mechanism is not very well elucidated. However, some mechanisms have been proposed. For example, the rupturing of the cell membrane and changes in the ion channels (Na^+^, K^+^, Ca^2+^, or Cl^−^) in the cell membrane might increase the permeability and cause the release of vital intracellular constituents [31], and inhibition of target enzymes [32]. Previous study confirm by scanning electron microscopy that the main mechanism of action of thymol is the membrane dysfunction, and suggests that thymol can be used as a naturally occurring drug against *S.* Typhimurium place of synthetic drugs [33]. The same result were verified for eugenol and terpineol for *S.* Typhimurium in Figure 4. Hydroxyl groups, such as those found in thymol, eugenol, terpineol and carvacrol, are highly reactive and form hydrogen bonds with active sites of target enzymes, inactivating them [32,34], and consequently, a dysfunction or rupture of the cell membrane. For example, thymol, which showed strong activity in the present work, is a compound commonly found in many essential oils and inhibits Gram-positive and Gram-negative bacteria, including *Bacillus subtilis*, *E. coli*, *Klebsiella pneumoniae* and *S. aureus* [3,26,35]. 

Friedman et al. [36], mentioned that essential oils and their compounds can be divided into two groups: slow acting compounds and fast-acting compounds. According to the results presented in Figure 1, it was observed that terpineol, eugenol, geraniol, carveol and citronellol were considered fast-acting compounds, since they inactivated organisms such as *E. coli* and *S.* Typhimurium in a short period (2 h). It has been reported that some antimicrobials considered fast-acting compounds are carvacrol, cinnamaldehyde and geraniol, since they inactivate organisms like *E. coli* and *S.* Typhimurium in five minutes, while compounds that act slowly requires 30–60 min to show efficient antimicrobial activity [36]. 

Previous studies verified the greater resistance of Gram-positive bacteria and mentioned that the greater resistance of Gram-positive cells may be due to their cell walls having a thick layer of peptidoglycan, making it difficult to pass antimicrobial agents and thus imparting rigidity to their cells [37,38].

The outer membrane of Gram-negative bacteria has porin channels, where the transport of low-molecular-weight substances occurs, and drugs with lipophilic characteristics have difficulty in crossing these channels [39]. In the present work, the compounds that showed the best activity in both MIC and time-kill kinetics have low molecular weights and polar functional groups. Such characteristics can increase antimicrobial capacity by facilitating penetration through the outer cell membrane. This hypothesis was verified by the action of eugenol, a phenolic compound of low molecular weight that presented fast time-kill kinetics, which led to the death of *S.* Typhimurium at all concentrations in only two h. In addition, Figure 3 and Figure 4 show that terpineol and eugenol affected the morphology of *S. aureus* and *S.* Typhimurium, respectively, indicating that the mechanism of action should be related by the rupture or dysfunction of the cell membrane. 

## 4. Materials and Methods

### 4.1. Materials

Terpene standards were obtained from Sigma-Aldrich (St. Louis, MO, USA). The structure of compounds are in Figure S1 (Supplementary Materials): (−)-α-bisabolol (95%), (−)-borneol (99%), (+)-borneol (97%), camphene (95%), (±) camphor (95%), carvacrol (98%), mixture of *cis* and *trans* l-carveol (95%), (+) carvone (98%), l-carvone (97%), β-caryophyllene (80%), citral (95%), (±) citronellal (95%), β-citronellol (95%), *m*-cymene (99%), *p*-cymene (99%), eucalyptol (99%), eugenol (99%), *trans*-geraniol (98%), guaiene (97%), α-humulene (96%), *R*-(+)-limonene (99%), (±)-linalool (97%), β-myrcene (100%), ocimene mixture of isomers (90%), α-phellandrene (75%), (+)-α-pinene (99%), (+)-β-pinene (98%), sabinene (75%), γ-terpinene (98%), terpineol, mixture of isomers (98%), terpinolene (90%), thymol (99%), (+)-valencene (65%). The bacterial strains *E. coli* (ATCC 8739), *S. aureus* (ATCC 25923), *S.* Typhimurium (ATCC 14028) and *B. cereus* (ATCC 14579) were obtained from the list of reference strains of INCQS-FIOCRUZ. DMSO (dimethylsulfoxide) was from Vetec (Rio de Janeiro, Brazil), and Mueller-Hinton agar (MH) and broth (MHB) were obtained from Himedia Laboratories PVT (Mumbai, India). The standard triphenyl tetrazolium chloride (TTC), sulfanilamide and the other reagents were purchased from Sigma-Aldrich.

### 4.2. Screening

The assay was performed according to the CLSI M7-A6 protocol [40]. The compounds were prepared in DMSO (0.75 mg/mL) in sterile Eppendorf and stored at 4 °C to minimize losses by volatilization. For the analyses, stock solutions were diluted in Mueller-Hinton broth and the final concentration of the compounds and DMSO in the well were 0.25 mg/mL and 0.25%, respectively. The inoculum was adjusted to there McFarland scale 0.5 (1.5 × 10^8^ CFU/mL) with a spectrophotometer (T80 +, PG Instruments, Leicestershire, UK) at 625 nm to reach an optical density of 0.08 to 0.10 and thereafter adjusted with Mueller Hinton broth so that each well of the microplate had 5 × 10^5^ CFU/mL. Negative control (Mueller-Hinton broth + DMSO 0.25% + inoculum) and sterility control (Mueller-Hinton broth with DMSO 0.25% without inoculum) were added to all plates, and the analyses were performed in triplicate. The assays were performed individually for each terpene and microorganism in order to avoid cross-interaction between the compounds by volatilization. Plates with lids were incubated at 35 °C for 24 h. After 24 h, 50 μL of TTC (0.5% aqueous solution) was added and then incubated at 35 °C for 6 h. The antimicrobial activity was verified by the inhibition of the visible growth of live cells, which was confirmed by TTC (dead cells are not colored). The compounds that presented positive activity in these results were selected for determining the minimum inhibitory concentrations (MICs).

### 4.3. Minimum Inhibitory Concentrations

The MIC determination was performed by the microdilution method according to the method M7-A6 of the CLSI [40]. The inoculum was adjusted to there McFarland scale 0.5 (1.5 × 108 CFU/mL) with a spectrophotometer (T80 +, PG Instruments, Leicestershire, UK) at 625 nm to reach an optical density of 0.08 to 0.10 and thereafter adjusted with Mueller Hinton broth so that each well of the microplate had 5 x 105 CFU/mL. The final concentration of the terpenes ranged from 0.25 to 0.002 mg/mL, the assays were performed individually for each terpene and microorganism to avoid cross-interaction between the compounds by volatilization. For every assay, the sterility control (Mueller-Hinton Broth with DMSO 0.25% without inoculum), the negative control (Mueller-Hinton broth with DMSO 0.25% and inoculum) and positive control (Mueller-Hinton broth with DMSO 0.25%, sulfanilamide and inoculum) were checked. All analyses were performed in triplicate. The plates were incubated at 35 °C for 24 h and sealed to minimize volatilization losses. After 24 h, 50 μL of TTC (0.5% aqueous solution) was added and then incubated at 35 °C for 6 h of incubation. The MIC of the compound was determined as being the lowest concentration of that compound that inhibited the visible growth of cells, which was confirmed by TTC (dead cells are not colored).

### 4.4. Minimal Bactericidal Concentration (MBC)

To determine minimum bactericidal concentrations (MBC), 100 µL aliquots from wells where no growth was observed (MIC, 2× MIC and 4× MIC) were plated in Petri dishes on Mueller-Hinton agar medium, which were then incubated in an oven at 35 °C for 24 h. MBC was defined as 99.9% (lack of growth) decrease in viable cells [41].

### 4.5. Time-kill Curve Studies 

The 24-hour time-kill curve study was performed according to the methodology described in document M26-A of the CLSI [42]. The concentrations evaluated were MIC, 2xMIC and 4× MIC and negative control (Mueller-Hinton broth with DMSO 0.25% and inoculum) were prepared. Bacterial suspension was prepared to obtain a turbidity comparable to 0.5 McFarland (1.5 × 10^8^ CFU/mL) that was adjusted to approximately 5 × 10^5^ CFU/mL after incubation into tube contains concentration MIC, 2xMIC and 4× MIC. These assay samples were incubated at 35ºC at 24h. A 10 μL aliquot of this homogenate was added to 990 μL of 0.9% sterile saline, and 100 μL of this solution was then added to Mueller-Hinton agar. The plating occurred at 0, 2, 4, 8, 12 and 24 h after preparation. The plates were incubated for 24 h at 35 °C. After incubation, the colonies were counted manually, and the obtained numbers were multiplied by 1000 to find the number of CFU/mL. These values were transformed to a logarithmic scale for the creation of time-of-death graphs. A reduction in the number of CFUs from the initial count by ≥ 3 log_10_ was defined as a bactericidal effect.

### 4.6. Scanning Electron Microscopy (SEM)

The procedure for SEM analysis was performed according previous study [43] with some modifications. *E. coli, S. aureus* and *S.* Typhimurium microorganisms were cultured on Mueller-Hinton agar. Mid-log-phase bacterial cultures were transferred to Mueller-Hinton broth and treated with different terpenes (terpineol, eugenol, carveol, citronellol and geraniol) within 4 h at a 4× MIC concentration. Suspensions of cells were fixed with a 2.5% glutaraldehyde solution and 0.1 M cacodylate buffer overnight. The samples were post-fixed with 1% osmium tetroxide for 40 minutes at room temperature. Samples were washed with 0.1 M cacodylate buffer and then dehydrated in a series of alcohol solutions at different concentrations, starting at 20% and increasing to 100% (*v*/*v*). The samples were then transferred to a sample basket and placed in a critical point dryer. The slides with samples were then coated with gold in a spray machine prior to their visualization with SEM.

## 5. Conclusions

Among the 33 evaluated compounds, the majority presented only bacteriostatic activity. The oxygenated terpenes showed strong antibacterial activity against all tested bacteria, especially Gram-negative bacteria. These compounds showed promising antimicrobials effects, even higher than sulfanilamide. The images obtained by SEM indicate that the mechanism of the cell death of the evaluated bacteria is due to loss of cellular membrane integrity or function. The present study brings detailed knowledge about the antimicrobial activity of the 33 individual compounds commonly present in essential oils, that can provide a greater understanding for the future researches of synergism, mechanism of action and bioavailability of different essential oils components. 

## Figures and Tables

**Table 1:** Antibacterial screening of compounds.

Compound	*B. cereus*	*S.* Typhimurium	*E. coli*	*S. aureus*
*Non-oxygenated monoterpenes*				
Camphene	−	−	−	−
*m*-Cymene	+	+	+	+
*p*-Cymene	−	−	−	−
Guaiene	−	−	−	−
R-(+)-Limonene	+	+	+	+
β -Myrcene	−	−	−	−
Ocimene	−	−	−	−
α-Phellandrene	−	−	−	−
(+)-α-Pinene	−	−	−	−
(+)-β-Pinene	−	−	−	−
Sabinene	−	−	−	−
Ƴ-Terpinene	−	−	−	−
Terpinolene	−	−	−	−
*Oxygenated monoterpene*				
(−)-α-Bisabolol	−	−	−	−
(−)-Borneol	+	+	+	+
(+)-Borneol	+	+	+	+
(±)-Camphor	+	+	+	+
l-Carveol	+	+	+	+
(+)-Carvone	−	−	−	−
l-Carvone	+	+	+	+
Citral	+	+	+	+
(±)-Citronellal	+	+	+	+
β-Citronellol	+	+	+	+
Eucalyptol	−	−	−	−
*trans*-Geraniol	+	+	+	+
(±)-Linalool	+	+	+	+
Terpineol	+	+	+	+
*Phenolic*				
Carvacrol	+	+	+	+
Eugenol	+	+	+	+
Thymol	+	+	+	+
*Sesquiterpene*				
β-Caryophyllene	−	−	−	−
α-Humulene	−	−	−	−
(+)-Valencene	−	−	−	−

(−) without activity; (+) with activity.

**Table 2:** Minimum inhibitory concentrations (mg/mL) of the compounds.

Compound	*B. cereus*	*S.* Typhimurium	*E. coli*	*S. aureus*
(−)-Borneol	0.12	0.12	0.25	0.03
(+)-Borneol	0.25	800	0.25	0.25
(±)-Camphor	0.25	0.25	0.25	0.015
Carvacrol	0.03	0.015	0.03	0.015
l-Carveol	0.12	0.03	0.06	0.015
l-Carvone	0.25	0.12	0.06	0.03
*m*-Cymene	0.25	0.25	0.25	0.25
Citral	0.06	0.07	0.06	0.06
Citronellal	0.12	0.12	0.25	0.25
β-Citronellol	0.12	0.12	0.25	0.03
Eugenol	0.07	0.07	0.03	0.003
*trans*-Geraniol	0.07	0.03	0.06	0.03
R-(+)-Limonene	0.25	0.06	0.25	0.25
Linalool	0.25	0.25	0.25	0.25
Terpineol	0.12	0.12	0.06	0.03
Thymol	0.007	0.003	0.007	0.007
Sulfanilamide	R	0.06	0.03	0.06

(R) Resistant.

**Table 3:** Minimum bactericidal concentrations (mg/mL) of the compounds.

Compound	*B. cereus*	*S.* Typhimurium	*E. coli*	*S. aureus*
l-Carveol	-	-	0.25	-
β-Citronellol	-	-	0.25	-
Eugenol	-	0.06	-	-
*trans*-Geraniol	-	-	0.25	-
Terpineol	-	0.25	-	0.12
Thymol	-	0.12	0.12	0.12

(-) bacterial growth.

## Supplementary Materials

The supplementary materials are available online.
