# Supplementary figures and images for: Antibacterial Activity of Terpenes and Terpenoids Present in Essential Oils
Source: Molecules. 2019 Jul 5;24(13):2471. doi: 10.3390/molecules24132471 (PMC6651100; doi:10.3390/molecules24132471)

# Supplementary Material

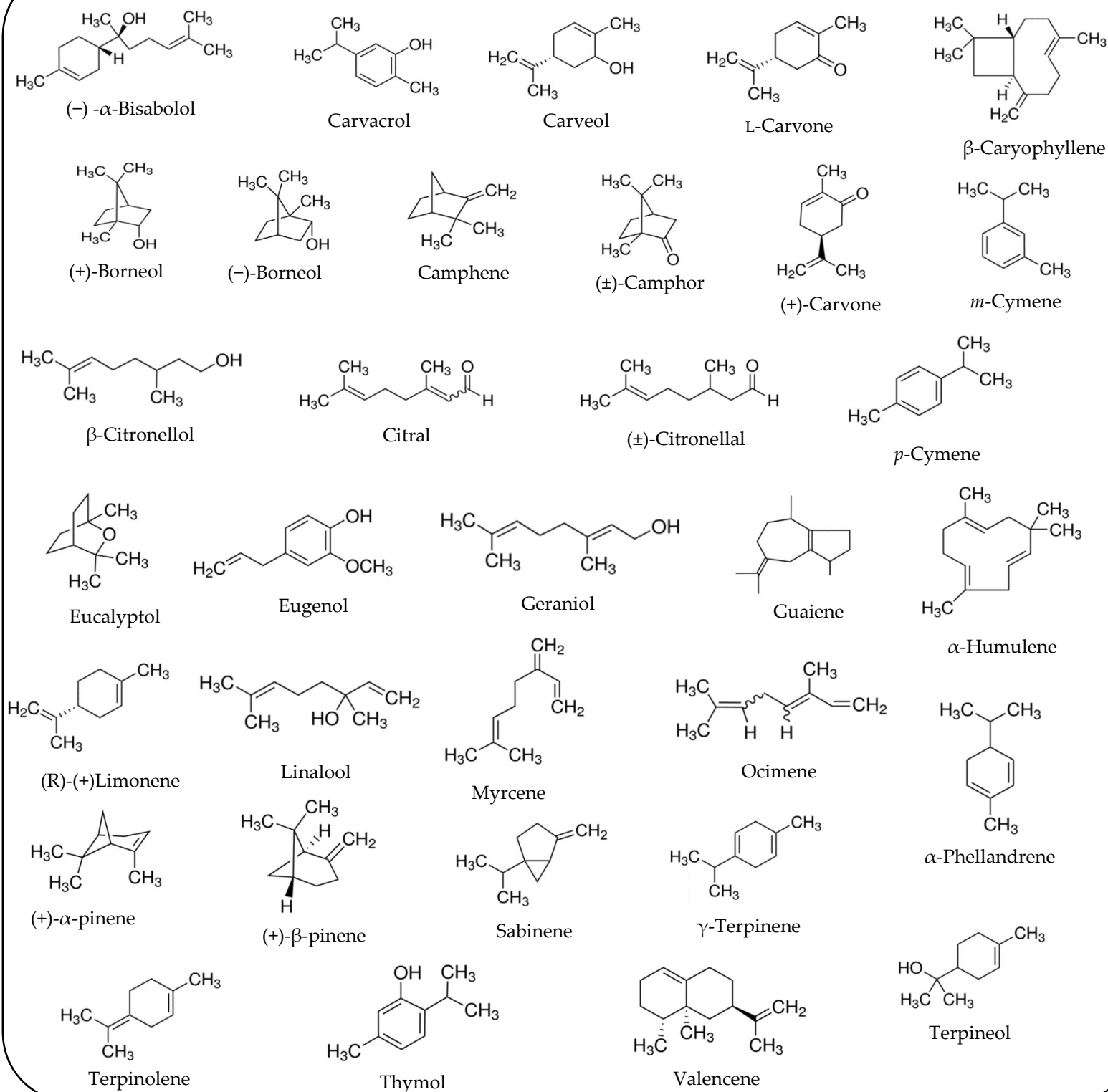

**Figure S1.** Structures list of the tested compounds.

Supplement: Supplementary file 1 [file molecules-24-02471-s001.pdf]
